# Efficacy of zinc sulfate in neonatal hyperbilirubinemia: a meta-analysis of randomized controlled trials

**DOI:** 10.3389/fped.2026.1703102

**Published:** 2026-05-18

**Authors:** Mohamed Saad Sayed, Shree Rath, Alaa Hamza Hermis, Arwa Mohamed Ibrahim, Sarshaar Qadir, Ahmad Omar Saleh, Sama Hesham Samir, Hafsa Shahid, Hiba Jasim Hafedh, Ursula Abu Nahla, Hebatullah Abdulazeem

**Affiliations:** 1 Faculty of Medicine, Beni-Suef University, Beni Suef, Egypt; 2 All India Institute of Medical Sciences, Bhubaneswar, India; 3 College of Nursing, University of Al-Qadisiyah, Al-Dewaniyah, Iraq; 4 College of Medicine, University of Sharjah, Sharjah, United Arab Emirates; 5 Shaikh Khalifa Bin Zayed Al-Nahyan Medical and Dental College, Lahore, Pakistan; 6 Faculty of Medicine, The University of Jordan, Amman, Jordan; 7 Faculty of Medicine, Helwan University, Cairo, Egypt; 8 Brigham and Women’s Health Hospital, HIM, Boston, MA, United States; 9 Faculty of Medicine, Hebron University, Hebron, Occupied Palestinian Territory; 10 Chair of Epidemiology, TUM School of Medicine and Health, Technical University of Munich, Munich, Germany

**Keywords:** hyperbilirubinemia, neonatal hyperbilirubinemia, phototherapy, zinc sulfate, zinc supplementation

## Abstract

**Background:**

Previous investigations into the impact of oral zinc sulfate on serum bilirubin levels of newborns have yielded conflicting results. This meta-analysis aimed to clarify the efficacy of zinc sulfate in infants with hyperbilirubinemia by combining the evidence from published randomized controlled trials (RCTs).

**Methods:**

In this systematic review, PubMed, Embase, Cochrane CENTRAL, Scopus, and Web of Science were searched in August 2024, with an update on 25 October 2025, for the RCTs investigating zinc sulfate in infants with hyperbilirubinemia. The primary outcome was the change in total serum bilirubin (mg/dL). Mean difference (MD) with 95% confidence interval [95% confidence interval (CI)] was used to estimate the overall effect of the outcomes. A random-effects model was used to pool the results.

**Results:**

A total of 14 RCTs comprising 1,405 infants were analyzed. Zinc sulfate significantly reduced bilirubin levels at 24 h [MD: −1.12 mg/dL, 95% CI (−1.73; −0.52), *p* = 0.0003, *I*^2^ = 90.5%], at 48 h [MD: −1.23 mg/dL, 95% CI (−2.19; −0.28), *p* = 0.0116, *I*^2^ = 89.1%], at 72 h [MD: −1.35 mg/dL, 95% CI (−2.57; −0.14), *p* = 0.0294, *I*^2^ = 95.4%], and at 4 days [MD: −1.57 mg/dL, 95% CI (−2.33; −0.81), *p* < 0.0001, *I*^2^ = 0.0%]. The high heterogeneity might be raised from the different clinical settings, such as gestational age, zinc sulfate doses, different therapy protocols among the regions and inconsistent phototherapy durations across the different regions. In the subgroup analysis, full-term infants with normal birth weights benefited from the intervention. On the other hand, no significant differences were observed in preterm and low-birth-weight infants. There were no significant differences between the zinc sulfate and placebo groups in phototherapy and hospital stay durations. GRADE assessment showed that most outcomes ranged from low to very low certainty of evidence.

**Conclusion:**

This meta-analysis showed that zinc sulfate may reduce total serum bilirubin levels in full-term, normal-birth-weight infants during the first 4 days with a modest effect. However, this conclusion is limited by the high heterogeneity and low certainty of evidence. There was no significant reduction in phototherapy and hospital stay durations.

## 1 Introduction

Hyperbilirubinemia is a global health problem in newborns, affecting approximately 60% of preterm babies and 80% of term babies (1). Elevated bilirubin levels are due to the imbalance between the production and clearance of bilirubin, which clinically manifests as jaundice (1, 2). Hyperbilirubinemia is caused by either the immature liver’s poor ability to conjugate the bilirubin or by increased RBC breakdown (1–3). Severe hyperbilirubinemia causes neurodevelopmental complications, which are extremely dangerous (4). With the high prevalence of hyperbilirubinemia, the associated risks, morbidity, mortality, and long-term neurodevelopmental issues, particularly in low- and middle-income countries, are particularly pronounced (4, 5).

The standard management of hyperbilirubinemia in neonates is phototherapy, which reduces bilirubin levels by converting unconjugated bilirubin into water-soluble molecules that the liver can excrete without conjugation (5–7). Phototherapy is effective in preventing progression to severe hyperbilirubinemia; however, prolonged use may cause complications such as temperature instability, retinal damage, dehydration, and reduced mother–infant contact during a critical bonding period (6–9). Some infants remain resistant to phototherapy, increasing their risk of severe hyperbilirubinemia and related complications despite adequate treatment (6, 9). In addition, in severe cases, exchange transfusion may be considered (10), but due to risks such as thrombocytopenia and hypocalcaemia, it is reserved for infants at high risk of bilirubin encephalopathy (10, 11). Intravenous immunoglobulins have been explored for iso-immune hemolytic jaundice due to ABO and Rh incompatibility (12), though they may increase the risk of necrotizing enterocolitis (13). These factors highlight the need for adjuvant treatment options to improve management and minimize prolonged therapy.

Recent literature has indicated that oral zinc supplementation (primarily zinc sulfate) can decrease serum unconjugated bilirubin in neonates by disrupting the enterohepatic circulation of unconjugated bilirubin (10, 11). In infants, the immature liver limits bilirubin conjugation, leading to its accumulation. Zinc may help in the reduction of bilirubin levels by modulating liver enzyme activity, enhancing conjugation and excretion, and inhibiting the heme oxygenase enzyme, which is the rate-limiting enzyme in bilirubin production (14). It also has antioxidant properties that may lower oxidative stress, which may be a contributing factor in neonatal hyperbilirubinemia. In full-term newborns, these effects may lead to a more pronounced decrease in serum bilirubin with zinc sulfate supplementation (10, 11). This supports the potential role of zinc as an adjuvant to phototherapy, shortening its duration and reducing complications. While previous meta-analyses by Yang et al. and Yadav et al. reported no significant benefit, they evaluated zinc sulfate for prophylaxis and therapeutic approaches (14, 15). More recent randomized controlled trials (RCTs) have assessed zinc sulfate as a therapeutic adjuvant, providing updated data on its efficacy in neonatal hyperbilirubinemia. Because of differences in bilirubin levels and current medications between high-risk newborns and affected neonates, we focused on studies that included affected neonates to assess the therapeutic role of zinc sulfate, while excluding studies that assessed the prophylactic role of zinc sulfate.

Based on this rationale, we hypothesized that oral zinc sulfate would accelerate bilirubin decline and reduce phototherapy duration compared with standard care alone. So, we aimed to analyze the effects of zinc sulfate in neonatal hyperbilirubinemia by pooling current studies focusing on the impact of zinc sulfate on bilirubin levels and phototherapy duration outcomes.

## 2 Methods

This systematic review and meta-analysis was conducted in adherence to the Cochrane Handbook (16) and reported according to the Preferred Reporting Items for Systematic Reviews and Meta-Analysis (PRISMA) guidelines (17). The predesigned protocol of this systematic review was registered in the International Prospective Register of Systematic Reviews (PROSPERO) database (under registration number PROSPERO ID: CRD42024583832).

### 2.1 Literature strategy

To evaluate the role of zinc sulfate in infants with hyperbilirubinemia undergoing phototherapy compared with placebo, we systematically searched PubMed, Embase, Cochrane Central Register of Controlled Trials, Scopus, and Web of Science on 15 August 2024, with an update on 25 October 2025. We searched through the databases by using mesh terms of zinc sulfate and hyperbilirubinemia including the following keywords: (“Zinc” OR “Zn” OR “Zinc Sulfate”) AND (“Neonatal Jaundice” OR “Neonatal Hyperbilirubinemia” OR “Neonatal Direct Hyperbilirubinemia” OR “Neonatal Indirect Hyperbilirubinemia”). No automated filters or language restrictions were applied. The detailed search strategy is provided in Supplementary Table 1. In addition, we performed a hand search using Google Scholar and the reference lists of included studies to identify any missed studies from the primary search. We also manually screened the ClinicalTrials.gov website.

### 2.2 Eligibility criteria and study selection

After searching databases, we removed duplicates of the retrieved records using EndNote 21. A preliminary title-and-abstract screening was performed by two independent reviewers (SR and SQ) Subsequently, eligible studies were screened by the same two reviewers (SR and SQ) via a full-text screening. Discrepancies during the screening process were resolved through discussion with a third reviewer (MSS). Interrater reliability was assessed using Cohen's *k* statistics, with values >0.80 interpreted as almost perfect agreement. The eligibility criteria were defined using the PICOS format:

- Population (P): studies including infants with hyperbilirubinemia, either full-term or preterm infants of any birth weight (includes low birth weight and normal birth weight).
- Intervention (I): studies in which any zinc sulfate was used.
- Comparison (C): studies comparing zinc sulfate with usual care (using phototherapy alone) or adding a placebo to usual care.
- Outcomes (O): change in reported total serum bilirubin (TSB) levels or phototherapy duration.
- Study design (S): RCTs.

Observational studies, conference papers, and review articles were excluded.

### 2.3 Data extraction

Two independent authors (MSS and SR) designed the data extraction sheets using Google spreadsheets. Four independent authors (SQ, SHS, AHH, and AMI) extracted the data, and MS solved any conflicts. The items extracted included study characteristics, population characteristics, and outcomes. The detailed items of each sheet are as follows:

- Overview of the study characteristics: study design, year of study, country, intervention details (dose and route of administration), comparison details, inclusion criteria, follow-up period, and study conclusion.
- Population characteristics: neonatal age (weeks), mean (SD); gestational age (weeks), mean (SD); male, *N* (%); weight (at birth), mean (SD); delivery vaginal, *N* (%),delivery cesarean *N* (%);TSB at baseline (mg/dL) mean (SD); hemoglobin level, g/dL, mean (SD); and reticulocyte count, mean (SD).
- Outcomes: (A) mean change in TSB levels across the follow-up period: first, second, third, and fourth day, (B) phototherapy duration (days), and (C) hospital stay (days).

### 2.4 Quality assessment and certainty of evidence

The quality of the studies included in this review was evaluated independently by two authors (SR and AMI) utilizing the Cochrane Risk of Bias Tool (ROB-2) (18). The assessment focused on five key domains: bias arising from the randomization process, bias due to deviations from intended interventions, bias due to missing outcome data, bias in measurement of the outcome, and bias in selection of the reported result. The authors' decisions were classified as “low risk of bias,” “high risk of bias,” or “some concerns.” Any conflicts between the two authors were resolved through discussion with a third author (MSS).

The GRADE criteria were used to determine the quality of the evidence (19). In summary, the overall quality of evidence was lowered if any of the following factors were present: risk of bias in the literature, inconsistency (high heterogeneity in the estimates), indirectness, imprecision [wide 95% confidence intervals (CIs) crossing the unity or failure to reach the optimal information size], and publication bias (19). The overall quality of the evidence supporting each outcome was graded as high, moderate, low, or extremely low after assessing each GRADE domain. GRADEpro GDT (GRADEpro Guideline Development Tool, McMaster University and Evidence Prime, 2024) was used for the GRADE assessment.

### 2.5 Result synthesis and data analysis

Estimates of each outcome were analyzed in the form of mean differences (MDs) with 95% confidence interval (95% CI). The restricted maximum likelihood model (REML) was used to perform the meta-analysis. Subgroup analyses were conducted according to zinc supplementation dose (some subgroups would be analyzed: 1, 1.2, 5 mg subgroup, 10 mg subgroup, and 20 mg subgroup), infant status (full-term or preterm), and weight at birth (normal weight or low birth weight).

Statistical heterogeneity between studies was evaluated using the chi-square test, Cochran's *Q* test, and *τ*² statistics. A chi-square *p*-value of less than 0.1 indicated significant heterogeneity and *I*^2^ value of ≥50% was considered high heterogeneity. The prediction interval was used to provide the clinical interpretation of heterogeneity. The leave-one-out model was used to resolve significant heterogeneity. Furthermore, for each outcome in the meta-analysis, sensitivity analyses were performed in several scenarios. In each scenario, one study was excluded to ensure that the overall impact size was unaffected by any single study. Sensitivity analyses were also conducted for the outcomes after excluding studies judged to have unclear or high risk of bias.

To assess publication bias in the analyzed outcomes, funnel plot analysis was applied as recommended by the Cochrane Handbook (16). Asymmetry may be inspected in the funnel plots, which may indicate the presence of small-study effects or publication bias. Egger's regression test was performed to statistically assess the asymmetry of the funnel plots, with significant asymmetry defined as a *p*-value of less than 0.10. All analyses were conducted using R version 4.1.1 (R Foundation for Statistical Computing, Vienna, Austria).

## 3 Results

### 3.1 Screening and study selection

**Figure 1:**
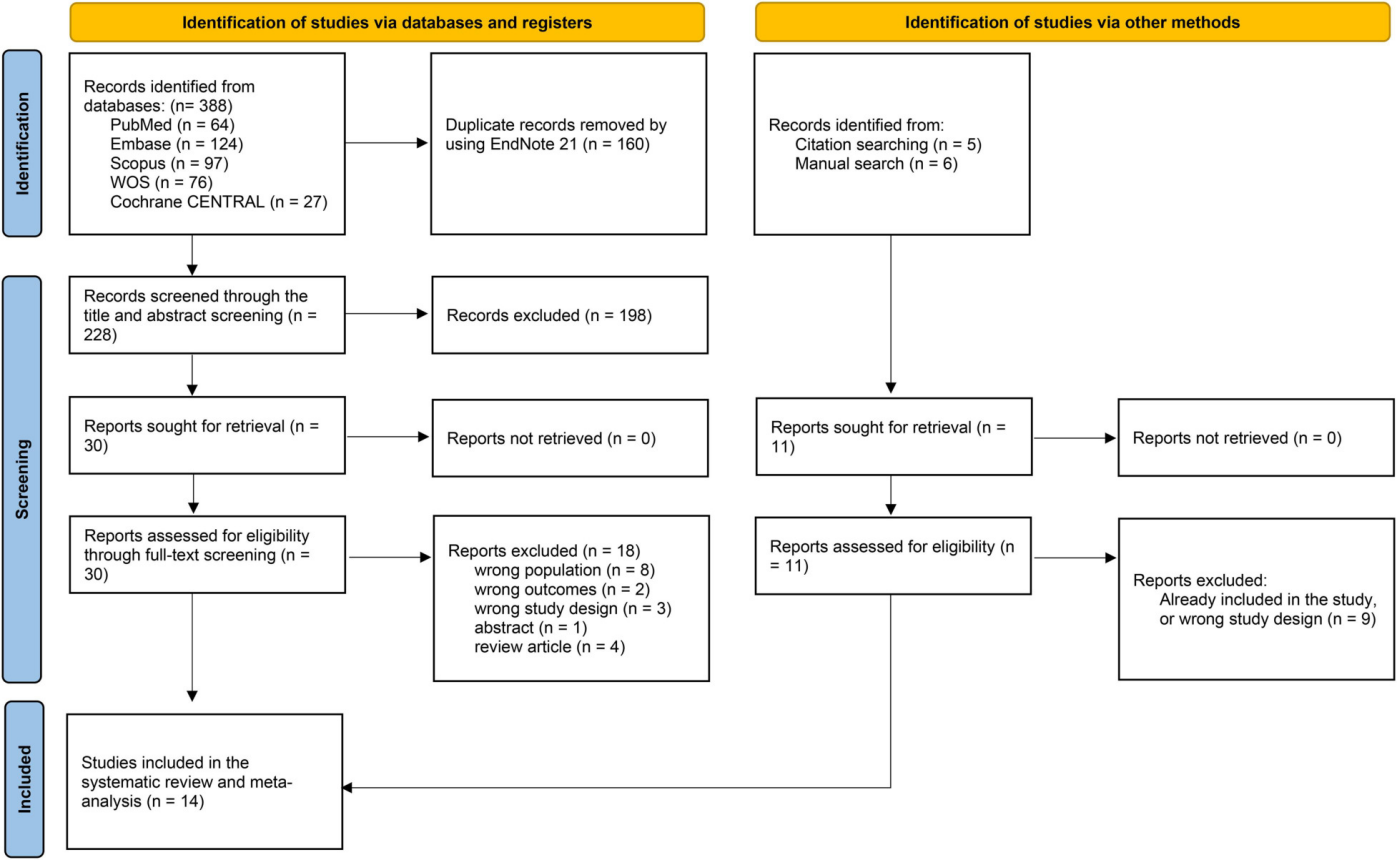
PRISMA flowchart for the literature search process through the databases.

From the detailed searches, 388 records were obtained, of which 288 were screened following the removal of duplicates. Thirty records were assessed for full-text screening, of which 12 studies were included. Reasons for exclusion included wrong population across five studies, wrong outcomes across two studies, wrong study design across three studies, one abstract, and four review articles. Reviewers demonstrated substantial agreement during title/abstract screening (*k* = 0.74) and almost perfect agreement during full-text screening (*k* = 0.85). An additional two studies were obtained through hand searching, resulting in a total of 14 studies included in the analysis (12, 13, 20–31). The PRISMA flowchart in Figure 1 presents the detailed screening steps.

### 3.2 Characteristics of included studies and patient characteristics

A total of 14 RCTs (12, 13, 20–31) were eligible, including a total patient population of 1,405 infants. These studies were conducted across multiple Southeast Asian and Middle Eastern countries, like Iran and India, with one study conducted in Egypt. Gestational age ranged from 31 to 38 weeks, with a mean gestational age of 36.3 weeks, comprising 63% boys and 47% girls. We excluded the study by Elfarargy et al. (20) from our analysis as it was a withdrawn preprint. A summary of all included studies is provided in Table 1. The baseline characteristics of the patients are provided in Table 2.

**Table 1:** Summary of included studies.

Study ID	Country	Study design/phase	Total sample size	Study period	Population	Intervention group				Control group	
						Name	Dose (day)	Route	Number in the group	Name	Number in the group
Mohammadzadeh (24)	Iran	RCT	60	From May 2013 to January 2014	Preterm with low birth weight	Zinc sulfate	20 mg/day	Oral	30	Phototherapy With placebo	30
Kumar (30)	India	RCT	80	From February 2008 to May 2009	Full term with Low birth weight	zinc sulfate	10 mg/day	Oral	40	phototherapy With placebo	40
Mohammadzadeh (21)	Iran	RCT	61	From May 2014 to May 24, 2015	preterm with low birth weight	Zinc sulfate	20 mg/day	Oral	30	Phototherapy With placebo	31
Ahmadpour-kacho (31)	Iran	RCT	105	June 2016 to February 2017	Full term with normal birth weight	Zinc sulfate	10 mg/day	Oral	50	Phototherapy alone	55
Hashemian (13)	Iran	RCT	66	July-December 2013	Full term with normal birth weight	Zinc sulfate	10 mg/day	Oral	33	Phototherapy with placebo	33
Beiranvand (28)	Iran	RCT	90	2013–2014	Full term with normal birth weight	Zinc sulfate	10 mg/day	Oral	45	Phototherapy alone	45
Faal (12)	Iran	RCT	60	March to June 2016	Preterm with low birth weight	Zinc sulfate	20 mg/day	Oral	30	Phototherapy with placebo	30
Eldesoky (23)	Egypt	RCT	100	February 2019 to February 2020	Full term	Zinc sulfate	10 mg/day	Oral	50	Phototherapy alone	50
Elfarargy (20)	Egypt	RCT	200	July 2016 to March 2018	Preterm with low birth weight	Zinc sulfate	10 mg/day	Oral	100	Phototherapy alone	100
Khoshnevisasl (29)	Iran	RCT	112	December 2017 to August 2018	Full term with normal birth weight	Zinc sulfate	10 mg/day	Oral	56	Phototherapy with Placebo	56
Mandlecha (26)	India	RCT	106	December 2017 to April 2019	Preterm with Low birth weight	Zinc sulfate	20 mg/day	Oral	53	Phototherapy with Placebo	53
Nikouei (27)	Kurdistan Province	RCT	290	Feb 2023 to Aug 2023	Full term, normal birth weight	Zinc sulfate	5 mg/day	Oral	160	Phototherapy with placebo	130
El-Araby (25)	Egypt	RCT	184	June 2018 to June 2019	Full term with some Low birth weight	Zinc sulfate	1 mg/kg	Oral	92	Phototherapy alone	92
Yeasin (22)	Bangladesh	RCT	60	January 2022 to November 2022	Full term, normal birth weight	Zinc sulfate + phototherapy	10 mg/day	Oral	30	Phototherapy alone	30

RCT, randomized controlled trial.

**Table 2:** Baseline characteristics for the patients in included studies.

Study ID	Intervention name	Neonates age (days), mean (SD)	Gestational age (weeks) mean (SD)	Male, *N* (%)	Weight (at birth) mean (SD)	Weight (at inclusion to study) g, mean (SD)	Delivery Vaginal, *N* (%)	Delivery Cesarean, *N* (%)	Total serum bilirubin at baseline (mg/dL) mean (SD)	Hemoglobin level, g/dL, mean (SD)	Reticulocyte count, mean (SD)
Mohammadzadeh (24)	Zinc sulfate	NA	31.8 (2.22)	10 (33.3%)	1,317.07 (169.09)	NA	2 (6.7%)	28 (93.3%)	6.89 (0.89)	NA	NA
	Placebo	NA	30.4 (2.04)	14 (46.7%)	1,260.33 (170.83)	NA	2 (6.7%)	28 (93.3%)	6.19 (1.37)	NA	NA
Kumar (30)	Zinc sulfate	88.80 (61.51)*	37.6 (1.5)	28 (70.0%)	2,711 (475)	2,595 (427)	NA	24 (60.0%)	13.9 (2.5)	NA	NA
	Placebo	85.83 (54.59)*	37.7 (1.4)	25 (62.5%)	2,672 (400)	2,554 (416)	NA	20 (50.0%)	13.4 (1.9)	NA	NA
Mohammadzadeh (21)	Zinc sulfate	6.07 (3.49)	34.06 (1.89)	11 (36.67%)	1,962.10 (315.45)	2,053 (364.18)	17 (58.1%)	13 (41.9%)	14.73 (3.22)	15.03 (2.75)	1.80 (1.41)
	Placebo	5.65 (5.29)	34.3 (2.48)	10 (32.3%)	2,053.00 (364.18)	1,962 (315.45)	16 (50%)	15 (50%)	14.87 (2.65)	15.92 (2.93)	1.51 (89)
Ahmadpour-kacho (31)	Zinc sulfate	5.8 (3.3)	NA	27 (54%)	NA	3,200 (420)	13 (26%)	36 (72%)	17.4 (2.1)	NA	NA
	Phototherapy	5.4 (1.6)	NA	29 (52.73%)	NA	3,100 (400)	13 (23.6%)	42 (76.4%)	17.1 (2.2)	NA	NA
Hashemian (13)	Zinc Sulfate	6.6 (2.57)	NA	19 (58.8%)	3,080.7 (564.60)	3,159.6 (489.37)	75%	NA	22.5 (2.31)	NA	1.3 (1.45)
	Phototherapy with placebo	5.8 (1.89)	NA	17 (52.8%)	3,155.2 (458.17)	3,173.0 (437.74)	52%	48%	21.5 (1.61)	NA	1.0 (1.01)
Beiranvand (28)	Zinc Sulfate and Phototherapy	NA	37.85 (1.17)	19 (42.2%)	3,080.22 (473.27)	2,964 (465.44)	NA	NA	15.08 (1.51)	16.11 (2.2)	NA
	Phototherapy Only	NA	38.14 (1.21)	23 (51.1%)	3,116.88 (406.86)	3,052 (379.21)	NA	NA	14.96 (1.26)	16.33 (2)	NA
Faal (12)	Zinc sulfate	70.21 (8.9)*	33.2 (1.27)	14 (56%)	NA	NA	NA	NA	NA	NA	NA
	Placebo	65.20 (6.8)*	32.1 (1.77)	11 (44%)	NA	NA	NA	NA	NA	NA	NA
Eldesoky (23)	Zinc sulfate	NA	NA	NA	NA	NA	NA	NA	16.98 (0.86)	NA	NA
	Phototherapy alone	NA	NA	NA	NA	NA	NA	NA	16.58 (1.31)	NA	NA
Elfarargy (20)	Zinc sulfate	NA	35.7 (0.6)	27 (54%)	NA	2,262 (95)	17 (34%)	33 (66%)	17.7 (1.1)	14.3 (0.7)	7.45 (0.58)
	Phototherapy only	NA	35.8 (0.5)	29 (58%)	NA	2,246 (112)	18 (36%)	32 (64%)	17.6 (1.2)	14.2 (0.8)	7.51 (0.49)
Khoshnevisasl (29)	Zinc sulfate	5 (1.24)	38.8 (1.03)	28 (50%)	3,223.8 (424.90)	NA	29 (51.8%)	27 (48.2%)	16.98 (3.33)	NA	NA
	Placebo	6 (1.24)	38.8 (0.98)	34 (60.7%)	3,165.5 (339.71)	NA	29 (51.8%)	28 (48.2%)	17.40 (3.24)	NA	NA
Mandlecha (26)	Zinc sulfate	92.7 (0.15)*	NA	26 (49)	2,809 (326.74)	NA	42 (79)	NA	19.11 (2.16)	15.01 (1.6)	2.69 (1.4)
	Placebo	87.85 (34.89)*	NA	30 (57)	2,755 (363.78)	NA	33 (62	NA	18.57 (2.14)	14.8 (1.13)	2.49 (1.5)
Nikouei (27)	Zinc sulfate	4.04 (2.56)	38.25 (1.05)	79 (49.38)	NA	3,124 (418.18)	NA	NA	16.65 (2.9)	NA	NA
	Placebo	4 (2.15)	38.27 (1.09)	66 (50.77)	NA	3,107.15 (441.69)	NA	NA	16.36 (2.96)	NA	NA
El-Araby (25)	Zinc sulfate	NA	37.33 (1.31)	46 (50)	NA	2,860 (520)	NA	NA	16.24 (3.01)	15.7 (2.16)	2.24 (0.54)
	Placebo	NA	37.31 (1.31)	48 (52.17)	NA	2,900 (460)	NA	NA	16.16 (2.83)	16.24 (2.57)	2.1 (0.6)
Yeasin (22)	Zinc sulfate + phototherapy	86.5 (19.8)*	38.2 (1.2)	13 (43.3%)	3,107.4 (430.7)	NA	NA	NA	16.7 (1.1)	16.44 (2.1)	NA
	phototherapy alone	87.2 (20.1)*	37.8 (1.3)	14 (46.7%)	3,133.2 (412.2)	NA	NA	NA	16.6 (1.2)	16.25 (2.2)	NA

* Age was reported by hours. NA: not available.

### 3.3 Quality assessment and certainty of evidence

**Figure 2:**
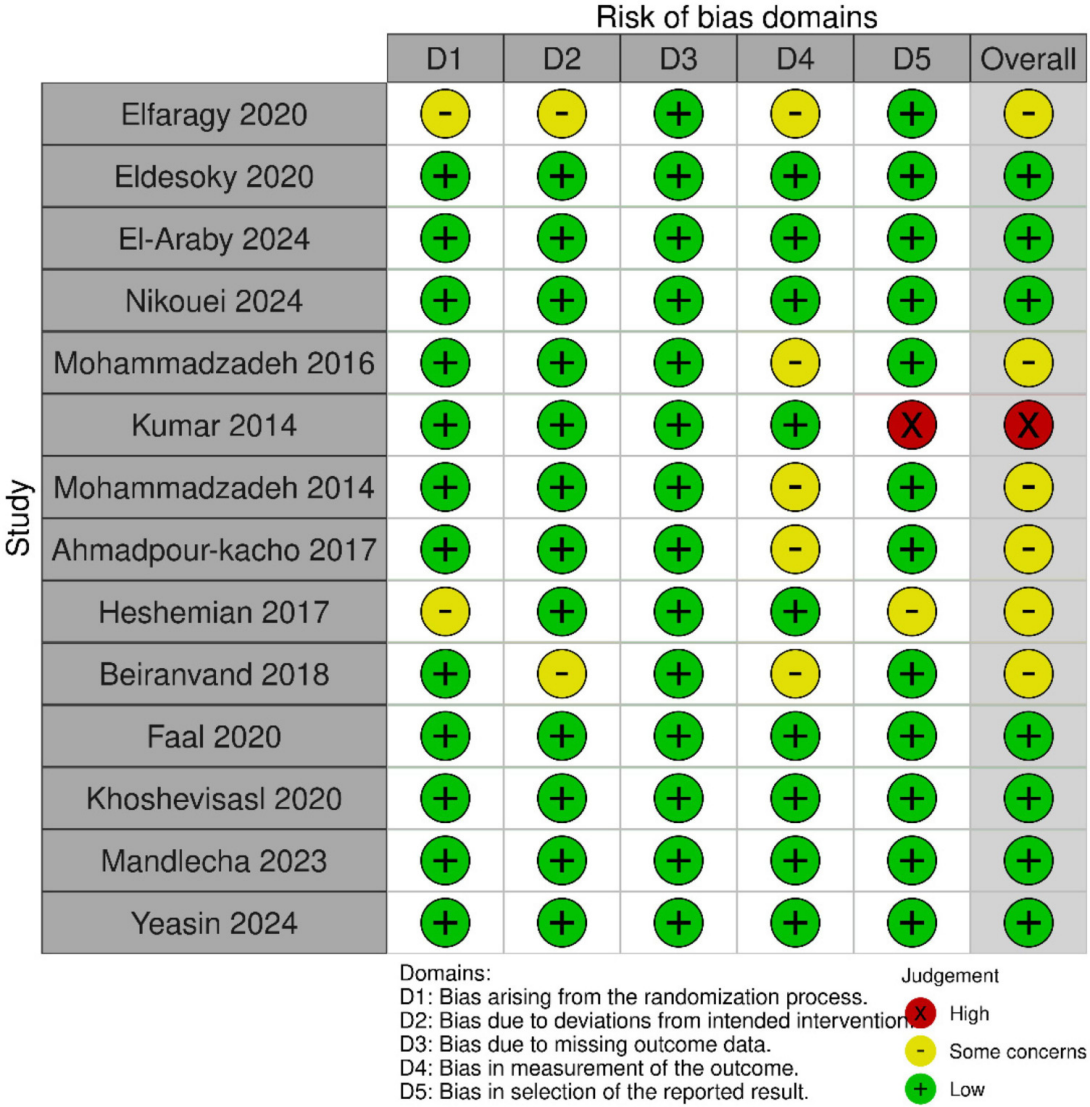
Risk-of-bias assessment for included studies.

The quality of studies was assessed using ROB 2. Six RCTs (13, 20, 21, 24, 28, 31) were judged showing some concerns, seven RCTs (12, 22, 25–27, 29) were low risk, and one RCT (30) showed high risk. The quality assessment graph and summary are presented in Figure 2. The studies by Elfarargy et al. (20) and Hashemian et al. (13) lacked sequence generation, with bias arising from the randomization process, and they showed “some concerns.” The studies by Elfarargy et al. (20) and Beiranvand et al. (28) showed bias due to deviations from intended interventions and were rated as showing “some concerns.” Four studies showed “some concerns” due to bias in the measurement of the outcome's domain. The study by Kumar (30) was at high risk of bias in reported outcomes. Certainty of analysis was determined following the GRADE assessment approach using GRADEpro. Change in TSB at 24 h was of moderate certainty due to the serious risk of bias and inconsistency in the studies. Change in TSB at 48 h and 4 days was rated as low certainty due to serious risk of bias and inconsistency, leading to downgrading of the evidence for both time points. Change in TSB at 72 h and phototherapy duration were rated as very low certainly because the risk of bias, inconsistency, and imprecision, leading to downgrading of the evidence. The GRADE assessment is summarized in Table 3. The heterogeneity details are presented in Supplementary Table 2.

**Table 3:** GRADE assessment of the analyzed outcomes.

Certainty assessment							No of patients		Effect		Certainty	Importance
No of studies	Study design	Risk of bias	Inconsistency	Indirectness	Imprecision	Other considerations	zinc	placebo	Relative (95% CI)	Absolute (95% CI)		
Change in total serum bilirubin (TSB) at 24 h												
11	Randomized trials	Not serious^a^	Serious^b^	Not serious	Not serious	None	579	550	-	MD **1.12 lower** (1.71 lower to 0.53 lower)	⊕⊕⊕◯ Moderate^a^^,^^b^	
Change in TSB at 48 h												
8	Randomized trials	Serious^c^	Serious^b^	Not serious	Not serious	None	439	381	-	MD **1.23 lower** (2.14 lower to 0.31 lower)	⊕⊕◯◯ Low^b^^,^^c^	
Change in TSB at 72 h												
8	Randomized trials	Serious^d^	Serious^b^	Not serious	Serious^e^	None	495	503	-	MD **1.28 lower** (2.75 lower to 0.19 higher)	⊕◯◯◯ Very low^b^^,^^d^^,^^e^	
Change in TSB at 4 days												
4	Randomized trials	Serious^f^	Serious^g^	Not serious	Not serious	None	191	197	-	MD **1.05 lower** (1.72 lower to 0.38 lower)	⊕⊕◯◯ Low^f^^,^^g^	
Phototherapy duration												
10	Randomized trials	Serious^h^	Serious^b^	Not serious	Serious^i^	None	564	537	-	MD **7.22 lower** (14.74 lower to 0.29 higher)	⊕◯◯◯ Very low^b^^,^^h^^,^^i^	

CI, confidence interval; MD, mean difference.

^a^ There were two studies with “some concerns” decisions and no downgrade level.

^b^ The point estimates suggest possible different inferences raised through high heterogeneity; subgroup analysis could not resolve heterogeneity. Downgraded one level.

^c^ There was one study with “some concerns” decisions; in addition, one study raised a “high risk.” Downgraded one level.

^d^ There were three studies with “some concerns” decisions that may affect the results of TSB at 72 h. Downgraded one level.

^e^ The upper boundary of the confidence interval (0.19) crossed the threshold of MD and included the trivial effect size. Downgraded one level.

^f^ There was one study that had “some concerns” and one study that had a “high risk” of bias, and this can affect the results. Downgraded one level.

^g^ The point estimates suggest possible different inferences raised through high heterogeneity; leave-one-out analysis showed that Ahmadpour-kacho affects the results. Downgraded one level.

^h^ There were two studies with “some concerns” decisions; in addition, one study raised a “high risk.” Downgraded one level.

^i^ The upper boundary of the confidence interval (0.29) crossed the threshold of MD and included the trivial effect size (Wide confidence interval). Downgraded one level.

### 3.4 Outcomes

#### 3.4.1 Mean change in TSB at different time points

##### 3.4.1.1 At 24 h

**Figure 3:**
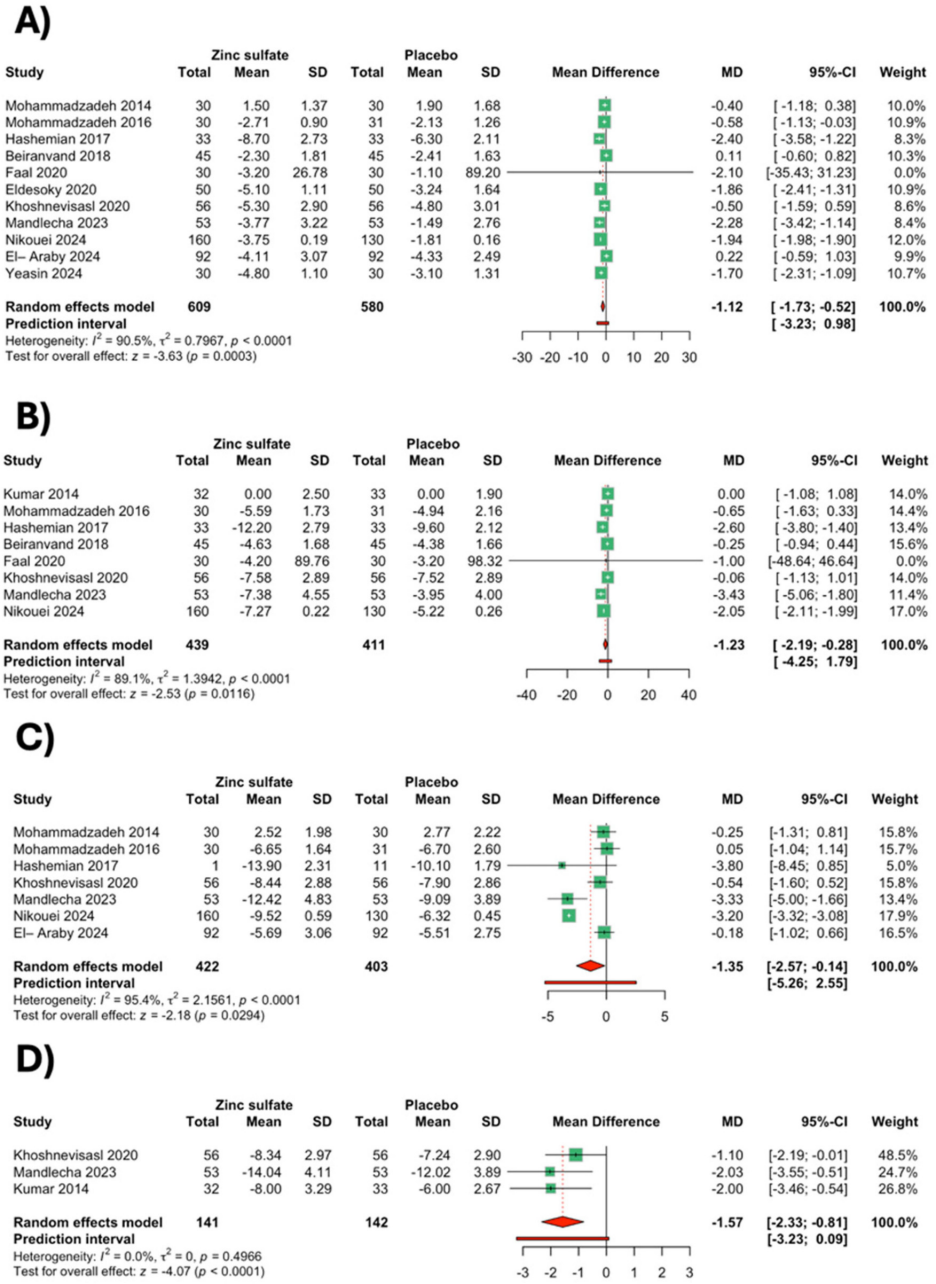
Meta-analysis forest plot for the mean change in total serum bilirubin (TSB) after zinc supplementation versus placebo, **(A)** after 24 h; **(B)** after 48 h; **(C)** after 72 h; and **(D)** after 4 days (random-effects meta-analysis). The numerical heterogeneity metrics were *I*² and *τ*² statistics. The prediction values are reported.

Mean change in TSB at 24 h after zinc sulfate administration was reported by 11 studies (12, 13, 21–29) involving 1,189 neonates (609 in the zinc sulfate group and 580 in the placebo group). Analysis showed that TSB was significantly reduced after zinc sulfate administration [MD: −1.12 mg/dL, 95% CI (−1.73; −0.52), *p* = 0.0003, *I*^2^ = 90.5%] (Figure 3A). A leave-one-out sensitivity analysis was attempted to assess the contribution of individual studies toward the pooled result. The analysis showed that results remained significant after excluding each study in the analysis (Supplementary Figure 1D). The sensitivity analysis after excluding unclear/high-risk studies showed that the results of the change in TSB at 24 h were consistent with the primary analysis [MD: −1.38 mg/dL, 95% CI (−2.13; −0.62), *p* = 0.0003, *I*^2^ = 82.8%] (Supplementary Figure 7A).

Subgroup analyses were performed based on the gestational age, weight of the neonates, and the dose of zinc sulfate administered. Zinc sulfate was associated with significant reduction in TSB levels at 24 h in both full-term neonates [MD: −1.39 mg/dL, 95% CI (−2.19; −0.59), *I*^2^ = 90.1%] and preterm neonates [MD: −0.52 mg/dL, 95% CI (−0.93; −0.10), *I*^2^ = 0%] (Supplementary Figure 1A). Zinc sulfate was associated with a significant reduction of TSB at 24 h in normal-birth-weight neonates [MD: −1.28 mg/dL, 9%CI (−2.22, −0.35), *I*^2^ = 87.6%] and low-birth-weight neonates [MD: −0.94 mg/dL, 95% CI (−1.74, −0.13), *I*^2^ = 91.1%] (Supplementary Figure 1B). It was noted that the TSB at 24  h was significantly reduced in the 1, 1.2, or 5 mg/day subgroup [MD: −1.94 mg/dL, 95% CI (−1.98, −1.90), *I*^2^ = 0%], 10 mg/day subgroup [MD: −1.01 mg/dL, 95% CI (−1.89; −0.13), *I*^2^ = 86.9%], and 20 mg/day subgroup [MD: −0.52 mg/dL, 95% CI (−0.97; −0.07), *I*^2^ = 0%] (Supplementary Figure 1C).

##### 3.4.1.2 At 48 h

Eight studies (12, 13, 21, 26–30) involving 850 neonates (439 in the zinc sulfate group and 411 in the placebo group) reported mean change in TSB at 48 h after zinc sulfate administration. The analysis showed that TSB was significantly reduced after zinc sulfate administration [MD: −1.23 mg/dL, 95% CI (−2.19; −0.28), *p* = 0.0116, *I*^2^ = 89.1%] (Figure 3B). Leave-one-out-sensitivity analysis showed that results were insignificant after excluding the study by Nikouei et al. [MD: −1.07 mg/dL, 95% CI (−2.17; 0.03)] (Supplementary Figure 2D). Sensitivity analysis after excluding unclear/high-risk studies showed that the results of the change in TSB at 48 h were inconsistent with the primary analysis [MD: −1.78 mg/dL, 95% CI (−3.59; 0.02), *p* = 0.053, *I*^2^ = 81.3%], highlighting the significant role of these studies in the effect estimate (Supplementary Figure 7B).

Subgroup analyses were performed based on gestational age and the dose of zinc sulfate administered. Based on the gestational age subgroup, TSB after 48 h significantly reduced in full-term neonates [MD: −1.59 mg/dL, 95% CI (−2.84; −0.34), *I*^2^ = 90.8%], but it was insignificant in preterm neonates [MD: −0.38 mg/dL, 95% CI (−1.10; 0.34), *I*^2^ = 0%] (Supplementary Figure 2A). TSB after 48 h was significantly reduced in normal-birth-weight neonates [MD: −1.99 mg/dL, 9%CI (−3.90, −0.08), *I*^2^ = 89.9%]; however, it was insignificant in the low-birth-weight neonates [MD: −0.79 mg/dL, 95% CI (−1.83, 0.24), *I*^2^ = 88.4%] (Supplementary Figure 2B). After analyzing studies based on dose, it was noted that the 1, 1.2, or 5 mg/day subgroup significantly reduced TSB after 48 h [MD: −2.49 mg/dL, 95% CI (−3.74; −1.23), *I*^2^ = 27.2%]. However, the use of 10 and 20 mg/day did not significantly reduce TSB levels (Supplementary Figure 2C).

##### 3.4.1.3 At 72 h

Seven studies (13, 20, 21, 24–27, 29) involving 825 neonates (422 in the zinc sulfate group and 403 in the placebo group) reported mean change in TSB at 72 h after zinc sulfate administration. The analysis showed that the mean change in TSB levels after 72 h had significantly reduced after zinc sulfate [MD: −1.35 mg/dL, 95% CI (−2.57; −0.14), *p* = 0.0294, *I*^2^ = 95.4%] (Figure 3C). Leave-one-out sensitivity analysis showed that results were insignificant after excluding studies by Hashemian et al., Mandlecha et al., or Nikouei et al. independently (Supplementary Figure 3D). Sensitivity analysis after excluding unclear/high-risk studies showed that the results of the change in TSB at 72 h were consistent with the primary analysis [MD: −1.79 mg/dL, 95% CI (−3.44; −0.13), *p* = 0.0343, *I*^2^ = 95.8%] (Supplementary Figure 7D).

Based on the gestational age subgroup, TSB after 72 h was significantly reduced in only the full-term neonates [MD: −2.36 mg/dL, 95% CI (−4.18; −0.55), *I*^2^ = 93.8%] but was insignificant in the preterm neonates [MD: −0.25 mg/dL, 95% CI (−0.87; 0.37), *I*^2^ = 0%] (Supplementary Figure 3A). The use of zinc sulfate in both low- and normal-weight infants was insignificant in decreasing the TSB at 72 h (*p* > 0.05) (Supplementary Figure 3B). After a subgroup of studies based on the dose, it was noted that 1, 1.2, or 5 mg/day significantly reduced the TSB after 72 h [MD: −3.20 mg/dL, 95% CI (−3.32; −3.08)]. However, the use of 10 and 20 mg/day did not significantly reduce TSB levels (*p* > 0.05) (Supplementary Figure 3C).

##### 3.4.1.4 At 4 days

Three studies (26, 29, 30) reported the TSB levels at 4 days after zinc sulfate administration (141 in the zinc group and 142 in the placebo group). The analysis showed that after zinc sulfate administration, the change in TSB levels was similar to the placebo group [MD: −1.57 mg/dL, 95% CI (−2.33; −0.81), *p* < 0.0001, *I*^2^ = 0.0%] (Figure 3D). Leave-one-out sensitivity analysis showed that results were consistent after exclusion of each study independently (Supplementary Figure 4A).

#### 3.4.2 Phototherapy duration

**Figure 4:**
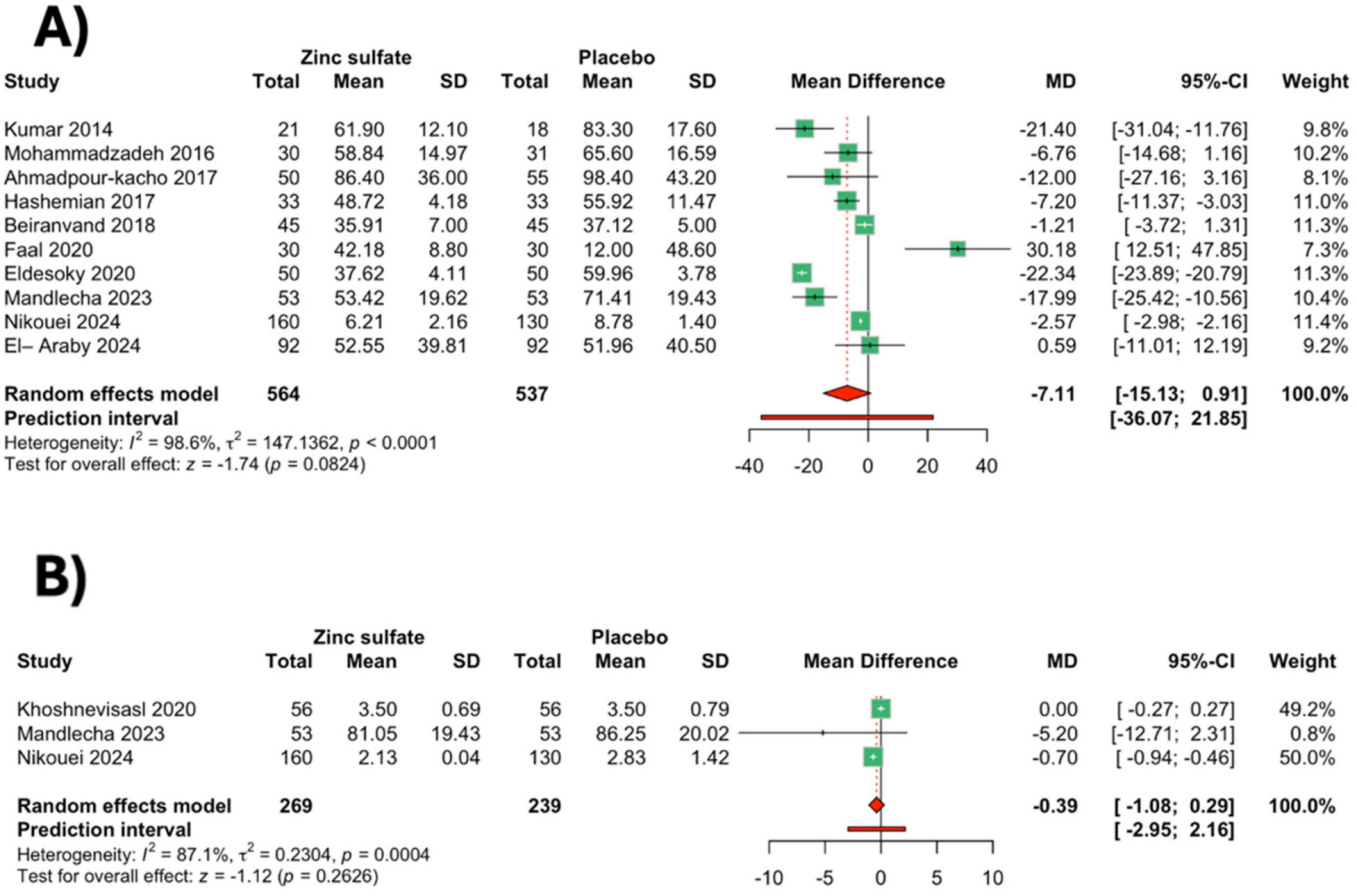
Meta-analysis forest plot for **(A)** mean change in the phototherapy duration; **(B)** mean change in the hospital stay duration (random-effects meta-analysis). The numerical heterogeneity metrics were *I*² and *τ*² statistics. The prediction values are reported.

A total of 10 studies (12, 13, 21, 23, 25–28, 30, 31) reported the change in phototherapy duration (564 in the zinc group and 537 in the placebo group). The analysis showed that phototherapy duration was similar between the two groups [MD: −7.11, 95% CI (−15.13; 0.91), *p* = 0.0824, *I*^2^ = 98.6%] (Figure 4A). Leave-one-out-sensitivity analysis was attempted to assess the contribution of individual studies toward the pooled result. The analysis showed that results were significant after the exclusion of a study by Faal et al. (12) [MD: −10.01, 95% CI (−15.97; −4.05)] (Supplementary Figure 5D). Sensitivity analysis after excluding unclear/high-risk studies showed that the results of the phototherapy duration were consistent with the primary analysis [MD: −3.66, 95% CI: (−20.60; 13.28), *I*^2^ = 99.3%] (Supplementary Figure 6D).

Subgroup analyses were performed based on the gestational age, weight of the neonates, and the dose of zinc sulfate administered. Phototherapy duration was significantly reduced in full-term neonates after zinc sulfate [MD: −10.42, 95% CI (−17.07; −3.76), *I*^2^ = 98.9%], but it was insignificant in the preterm neonates [MD: 10.83, 95% CI (−25.33; 46.99), *I*^2^ = 92.8%] compared with placebo (Supplementary Figure 5A). The phototherapy duration was significantly reduced after zinc sulfate in the normal-birth-weight infants [MD: −10.31, 95% CI (−18.10; −2.51), *I*^2^ = 97.8%] compared with placebo; however, it was similar between the two groups in the low-birth-weight infants (Supplementary Figure 5B). The dose of 10 mg/day of zinc sulfate was associated with a lower phototherapy duration [MD: −10.70, 95% CI (−18.93; −2.47), *I*^2^ = 97.8%] compared with placebo; however, phototherapy duration was similar in the 1, 1.2, or 5 mg/day subgroup and the 20 mg/day subgroup (Supplementary Figure 5C).

#### 3.4.3 Hospital stay (days)

A total of three studies (26, 27, 29) were included in the analysis, involving 508 infants. There was no significant difference between the zinc sulfate and placebo groups in the reduction of hospital stay duration [MD: −0.39, 95% CI (−1.08; 0.29), *p* = 0.26, *I*^2^ = 87.1%] (Figure 4A). To address the source of heterogeneity, a sensitivity leave-one-out analysis was done. The results remain insignificant after the exclusion of each study from the analysis (Supplementary Figure 4B).

#### 3.4.4 Publication bias

**Figure 5:**
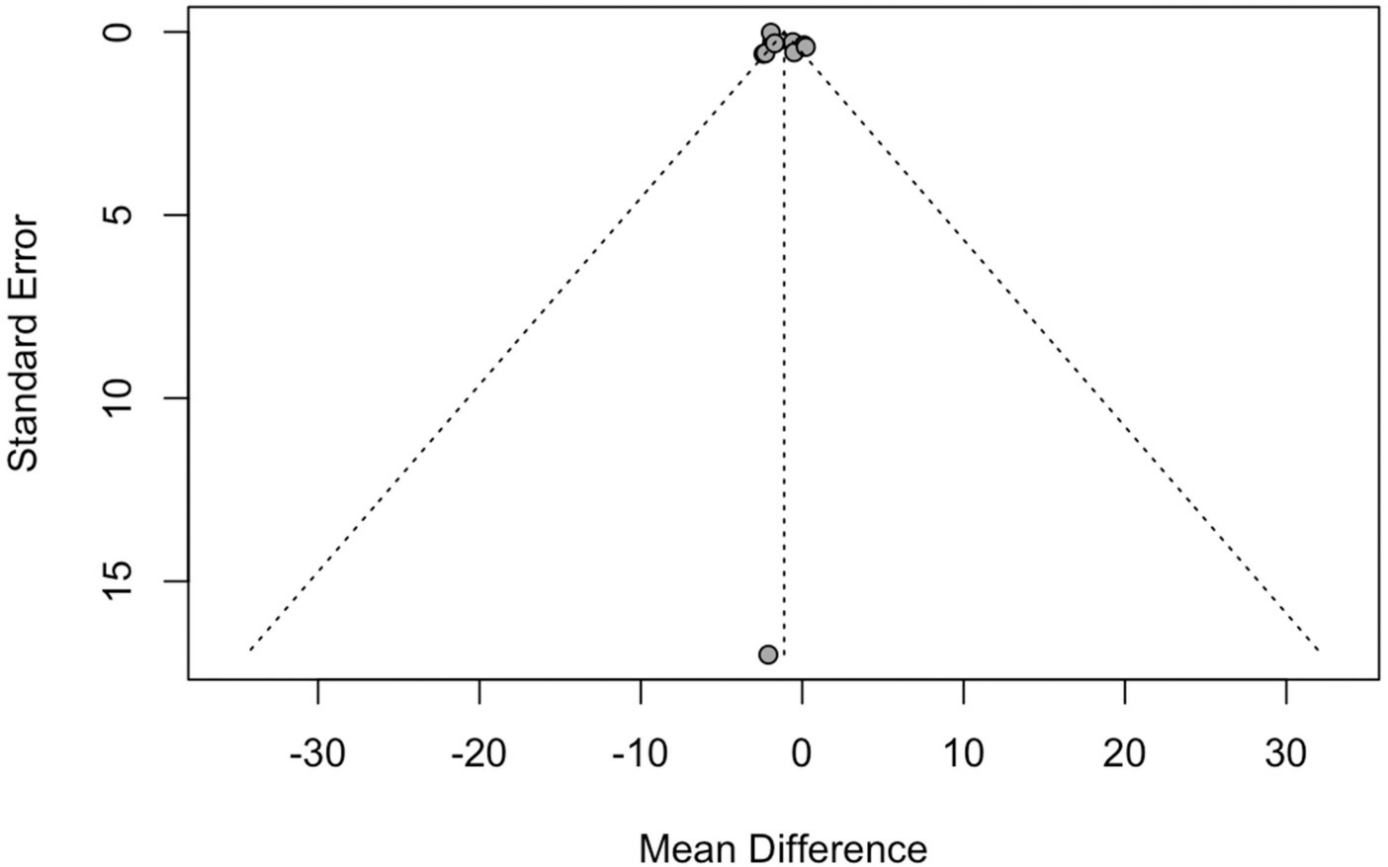
Funnel plot for publication bias (TSB at 24 h). The dashed line reflects the pseudoconfidence limits.

A funnel plot was generated to assess publication bias for the mean change in TSB at 24 h. The plot showed asymmetry, with most of the studies at the top of the plot, suggesting the presence of publication bias (Figure 5). Egger's test revealed no publication bias in this outcome (small study effects) (*p* = 0.98).

## 4 Discussion

This comprehensive meta-analysis gathered data through RCTs to evaluate the role of zinc sulfate administration in the reduction of TSB levels and phototherapy duration (days) in infants with hyperbilirubinemia. Based on 14 RCTs (12, 13, 20–31) including 1,405 infants, zinc sulfate administration significantly reduced TSB up to 4 days after administration with moderate effects and low certainty of evidence. Full-term babies showed a significant reduction in TSB levels after 48 and 72 h of zinc sulfate administration; the reduction was very low. However, preterm babies showed no reduction in TSB levels. Based on term status, there was a significant reduction in the phototherapy duration in full-term infants compared with preterm infants after administration of zinc sulfate. The weight status did not affect the duration between the two groups. Only the 10 mg/day zinc sulfate dose significantly reduced the duration of phototherapy compared with the placebo; however, other doses did not. Particularly in full-term infants, the use of zinc sulfate in the first 2 days could reduce bilirubin levels and the duration of phototherapy. In addition, sensitivity and subgroup analyses suggested using lower doses of zinc sulfate in full-term infants for 3 days. Overall, the certainty of evidence for bilirubin reduction was moderate to very low, warranting caution when interpreting our results.

These findings align with previous studies exploring zinc's role in neonatal hyperbilirubinemia. The updated meta-analysis by de Oliveira et al. reported that zinc sulfate significantly reduced bilirubin levels at 24, 48, and 72 h, particularly in term neonates (MD = −0.76 mg/dL at 24 h; MD = −1.19 mg/dL at 72 h) (32). This aligns with this review's results, which demonstrated a significant reduction at 24, 48, 72 h, and 4 days. Discrepancies in the results compared with the previous meta-analysis may stem from differences in calculating the mean change in TSB, which in this study was adjusted for baseline differences in bilirubin levels. Significant bilirubin decreases and shorter phototherapy times are linked to a dosage of 5 mg per day. Higher dosages, however, have inconsistent outcomes, possibly because of altered bioavailability or saturation effects (24). In contrast to these results, a meta-analysis conducted by Yang et al. concluded that zinc sulfate did not significantly reduce bilirubin levels at 3 and 7 days but decreased the phototherapy duration (14). This differs from the present meta-analysis findings, which showed no significant reduction in phototherapy duration except in specific subgroups. Variations may be due to the inclusion of recent studies conducted after 2017. However, these findings aligned with the results of Yang et al., which were significant after the leave-one-out was completed, particularly after the exclusion of the study by Faal et al. (12) [MD: −10.01].

These results are consistent with a previous meta-analysis that evaluated zinc sulfate as a therapeutic intervention (32, 33). However, they differ from the study that combined the therapeutic and prophylactic intervention usage of zinc sulfate within the same analysis (14). Importantly, there were many baseline characteristic differences in serum bilirubin levels at admission across the included studies. Therefore, the mean change was calculated between the bilirubin levels at baseline of zinc sulfate administration, and the time point of outcome measurement to adjust for baseline differences. The possible mechanism underlying zinc’s effect could be its inhibiting of enterohepatic circulation of unconjugated bilirubin, as zinc salts precipitate unconjugated bilirubin in the intestinal lumen, reducing its reabsorption (32, 34). Moreover, zinc may decrease intestinal absorption of bilirubin by direct binding, further limiting its enterohepatic recycling. This contrasts with the approach of de Oliveira et al. (32), who analyzed the raw data at each time point without adjusting for the differences between the bilirubin levels at baseline. Reductions in TSB levels were low with moderate to very low certainty of evidence because of both high heterogeneity and wide 95% CIs. The high heterogeneity might be due to the clinical heterogeneity (differences in baseline TSB, zinc sulfate dose, or gestational age).

The subgroup analyses revealed that zinc sulfate was more effective in reducing TSB in full-term neonates than in preterm neonates. This is consistent with other research indicating that the therapeutic effectiveness of zinc sulfate may be impacted by variations in zinc metabolism, depending on gestational age (33, 35). This is because full-term infants have more mature intestinal functions and bile acid pools, facilitating zinc–bilirubin interactions in the gut and more efficient interruption of enterohepatic circulation. In contrast, preterm infants' immature gut, altered microbiome, and slower intestinal transit likely blunt this effect (32, 33). It should be noted that the reduction after 1 mg/dL zinc sulfate is not clinically meaningful enough to include it in the treatment protocol.

Many complications and adverse effects have arisen from using phototherapy on infants, and these complications may disrupt the normal development of babies (6–8). Thus, reductions in phototherapy, particularly in some subgroups, such as full-term babies—and after excluding some studies that raised some heterogeneity concerns, such as Faal et al. (12)—are clinically important. It is also crucial to highlight that the mean change in phototherapy duration was insignificant in our analysis, but there was high heterogeneity in this outcome. This may be due to different physiotherapy protocols and methods. Sensitivity analyses, excluding the unclear/high-risk studies, revealed consistent results with the primary analysis. Notably, the mean change in phototherapy duration was significant after excluding the study by Faal et al. (12), although it was included in our analysis as its methodology was sound. The different clinical decisions between the practitioners in the clinical practice and the various thresholds to phototherapy discontinuation may contribute to the insignificance and inconsistencies in the phototherapy duration between the reported studies. The absence of standard guidelines across institutions makes the phototherapy discontinuation decision a subjective process. Moreover, regional differences may influence outcomes after zinc sulfate administration.

This study's strengths include its comprehensive approach, gathering all available RCTs to provide a robust and precise assessment of zinc sulfate's efficacy in neonatal hyperbilirubinemia. Hospital stay duration is a crucial patient-centered outcome. We employed the random-effects model, along with sensitivity and subgroup analyses, to test the effect size of each analysis. Notably, this study has several limitations. First, high heterogeneity was observed in some outcomes, likely due to variations in population characteristics (term status and gestational age), birth weight of neonates, zinc sulfate doses, and zinc sulfate administration protocols in the included studies. Second, the lack of standardization in phototherapy protocols across studies and the small sample sizes of studies were important limitations. To address it, sensitivity analyses through leave-one-out analysis were conducted to explore each study's effect on the overall effect estimate. Third, subgroup analyses, besides using the REML model, were performed. A limited number of included studies reported outcomes in the specific subgroups of preterm babies and low-birth-weight infants, underpowering the interpretation and making the generalizability of these results questionable. So, the results of subgroup analyses should be interpreted with caution. Fourth, a limited number of studies reported hospital stay, highlighting that this outcome needs to be addressed in upcoming studies. Fifth, the subgroup analysis based on infant sex was not feasible because the included RCTs did not report bilirubin changes separately by sex. Sixth, the search was limited to published studies and new studies on ClinicalTrials.gov, and gray literature was not searched, raising potential publication bias. Moreover, the included studies lacked details on the types of phototherapies used, such as light wavelength and irradiance, so we could not perform any subgroup analysis based on phototherapy. Seventh, Egger's test was applied to fewer than 10 studies, limiting its power, so its results should be interpreted with caution. Eighth, low to very low certainty of evidence was observed across the analyzed outcomes.

Our findings extend beyond statistics, offering practical insights into zinc's potential as an adjunct therapy in neonatal hyperbilirubinemia management. In addition to the protocols of hyperbilirubinemia management such as phototherapy, using zinc sulfate can achieve good results and may shorten phototherapy duration, particularly in full-term babies. However, large, well-designed RCTs are needed to study the effect of zinc sulfate, stratified by gestational age and birth weight, with standardized zinc dosing and safety monitoring. Our conclusions are limited to small studies that reported outcomes after 20 mg/day. Moreover, the 1 or 1.2 mg/day effects across the short-term period do not justify the clinical decision to utilize small doses as treatment. Addressing hospital stay duration is necessary in future RCTs because of its importance for both patients and healthcare authorities.

## 5 Conclusion

The analysis demonstrated that zinc sulfate may reduce TSB levels in neonates, particularly within the first 4 days of treatment. However, the certainty of evidence was low and heterogeneity was high. Full-term neonates appeared to experience modest benefits in phototherapy reduction, while zinc sulfate did not consistently reduce phototherapy duration, with high heterogeneity in this outcome. Future research should prioritize optimal dosing strategies, evaluate special subgroups such as preterm and low-birth-weight infants, and report hospital stay outcomes.

## Data Availability

The original contributions presented in the study are included in the article/Supplementary Material, further inquiries can be directed to the corresponding author.
