# Supplementary material for: Efficacy of zinc sulfate in neonatal hyperbilirubinemia: a meta-analysis of randomized controlled trials
Source: Front Pediatr. 2026 May 18;14:1703102. doi: 10.3389/fped.2026.1703102 (PMC13223103; doi:10.3389/fped.2026.1703102)
Supplement: Supplementary file 1 [file Supplementaryfile1.docx]

**SUPPLEMENTARY APPENDIX**

| Database | Search terms | Filters | Date of search | Records |
| --- | --- | --- | --- | --- |
| PubMed | ("Zinc" OR "Zn" OR "Zincteral" OR "Zinc Sulfate, Heptahydrate") AND ("Neonatal Jaundice" OR "Physiological Neonatal Jaundice" OR "Severe Jaundice in Newborns" OR "Icterus Gravis Neonatorum" OR "Severe Jaundice in Neonate" OR "Hyperbilirubinemia During Infancy" OR "During Infancies, Hyperbilirubinemia" OR "Neonatal Hyperbilirubinemia" OR "Neonatal Direct Hyperbilirubinemia" OR "Indirect Hyperbilirubinemia, Neonatal" OR "Neonatal Indirect Hyperbilirubinemia") | ALL fields | 25 October 2025 | 64 |
| Embase | ("Zinc" OR "Zn" OR "Zincteral" OR "Zinc Sulfate, Heptahydrate") AND ("Neonatal Jaundice" OR "Physiological Neonatal Jaundice" OR "Severe Jaundice in Newborns" OR "Icterus Gravis Neonatorum" OR "Severe Jaundice in Neonate" OR "Hyperbilirubinemia During Infancy" OR "During Infancies, Hyperbilirubinemia" OR "Neonatal Hyperbilirubinemia" OR "Neonatal Direct Hyperbilirubinemia" OR "Indirect Hyperbilirubinemia, Neonatal" OR "Neonatal Indirect Hyperbilirubinemia") | Title, abstract, keywords | 25 October 2025 | 124 |
| Scopus | ("Zinc" OR "Zn" OR "Zincteral" OR "Zinc Sulfate, Heptahydrate") AND ("Neonatal Jaundice" OR "Physiological Neonatal Jaundice" OR "Severe Jaundice in Newborns" OR "Icterus Gravis Neonatorum" OR "Severe Jaundice in Neonate" OR "Hyperbilirubinemia During Infancy" OR "During Infancies, Hyperbilirubinemia" OR "Neonatal Hyperbilirubinemia" OR "Neonatal Direct Hyperbilirubinemia" OR "Indirect Hyperbilirubinemia, Neonatal" OR "Neonatal Indirect Hyperbilirubinemia") | Title, abstract, keywords | 25 October 2025 | 97 |
| WOS | ("Zinc" OR "Zn" OR "Zincteral" OR "Zinc Sulfate, Heptahydrate") AND ("Neonatal Jaundice" OR "Physiological Neonatal Jaundice" OR "Severe Jaundice in Newborns" OR "Icterus Gravis Neonatorum" OR "Severe Jaundice in Neonate" OR "Hyperbilirubinemia During Infancy" OR "During Infancies, Hyperbilirubinemia" OR "Neonatal Hyperbilirubinemia" OR "Neonatal Direct Hyperbilirubinemia" OR "Indirect Hyperbilirubinemia, Neonatal" OR "Neonatal Indirect Hyperbilirubinemia" | Title, abstract, keywords | 25 October 2025 | 76 |
| Cochrane CENTRAL | ("Zinc" OR "Zn" OR "Zincteral" OR "Zinc Sulfate, Heptahydrate") AND ("Neonatal Jaundice" OR "Physiological Neonatal Jaundice" OR "Severe Jaundice in Newborns" OR "Icterus Gravis Neonatorum" OR "Severe Jaundice in Neonate" OR "Hyperbilirubinemia During Infancy" OR "During Infancies, Hyperbilirubinemia" OR "Neonatal Hyperbilirubinemia" OR "Neonatal Direct Hyperbilirubinemia" OR "Indirect Hyperbilirubinemia, Neonatal" OR "Neonatal Indirect Hyperbilirubinemia") | Title, abstract, keywords | 25 October 2025 | 27 |

**Supplementary Table 1:** Detailed search strategy for each database.

| Outcome | I² | τ² | Overall GRADE |
| --- | --- | --- | --- |
| Change of TSB at 24 h | 90.5% | 0.79 | Moderate |
| Change of TSB at 48 h | 89.1% | 1.39 | Low |
| Change of TSB at 72 h | 95.4% | 2.15 | Very Low |
| Change of TSB in 4 days | 0.0% | 0 | Low |
| Phototherapy duration | 98.6% | 147.13 | Very Low |

**Supplementary Table 2:** Heterogeneity summary table including heterogeneity statistics and overall GRADE assessment for the outcomes.


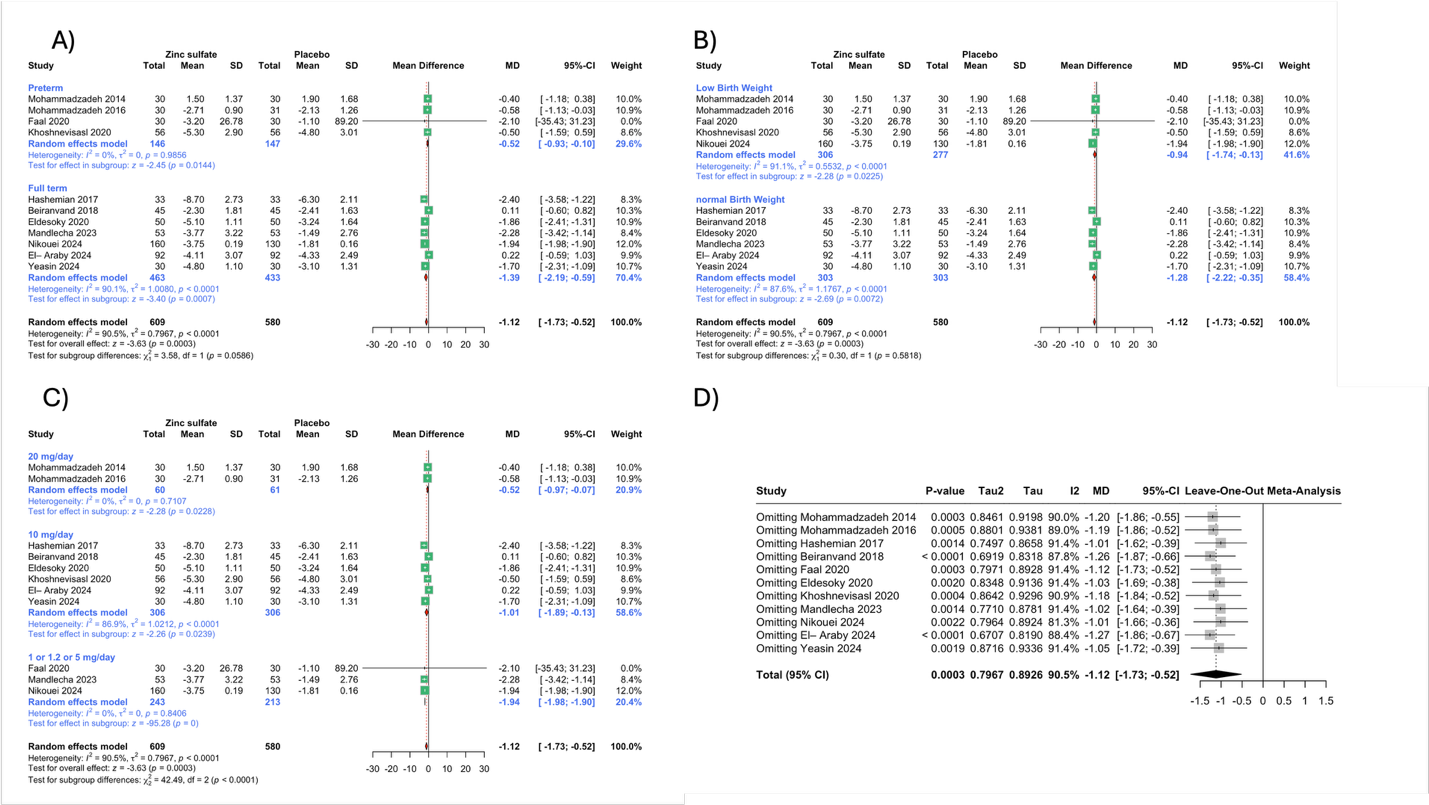


**Supplementary Figure 1:** Meta-analysis forest plot for the change in total serum bilirubin (TSB) at 24 hours. **A**) Subgrouping according to term status. **B**) Subgrouping according to birth weight. **C**) Subgrouping according to zinc dosage. **D**) Sensitivity Leave-one-out for change of total serum bilirubin (TSB) at 24 hours (**Random effects Model). The numerical heterogeneity metrics were I² and τ² statistics.**

**
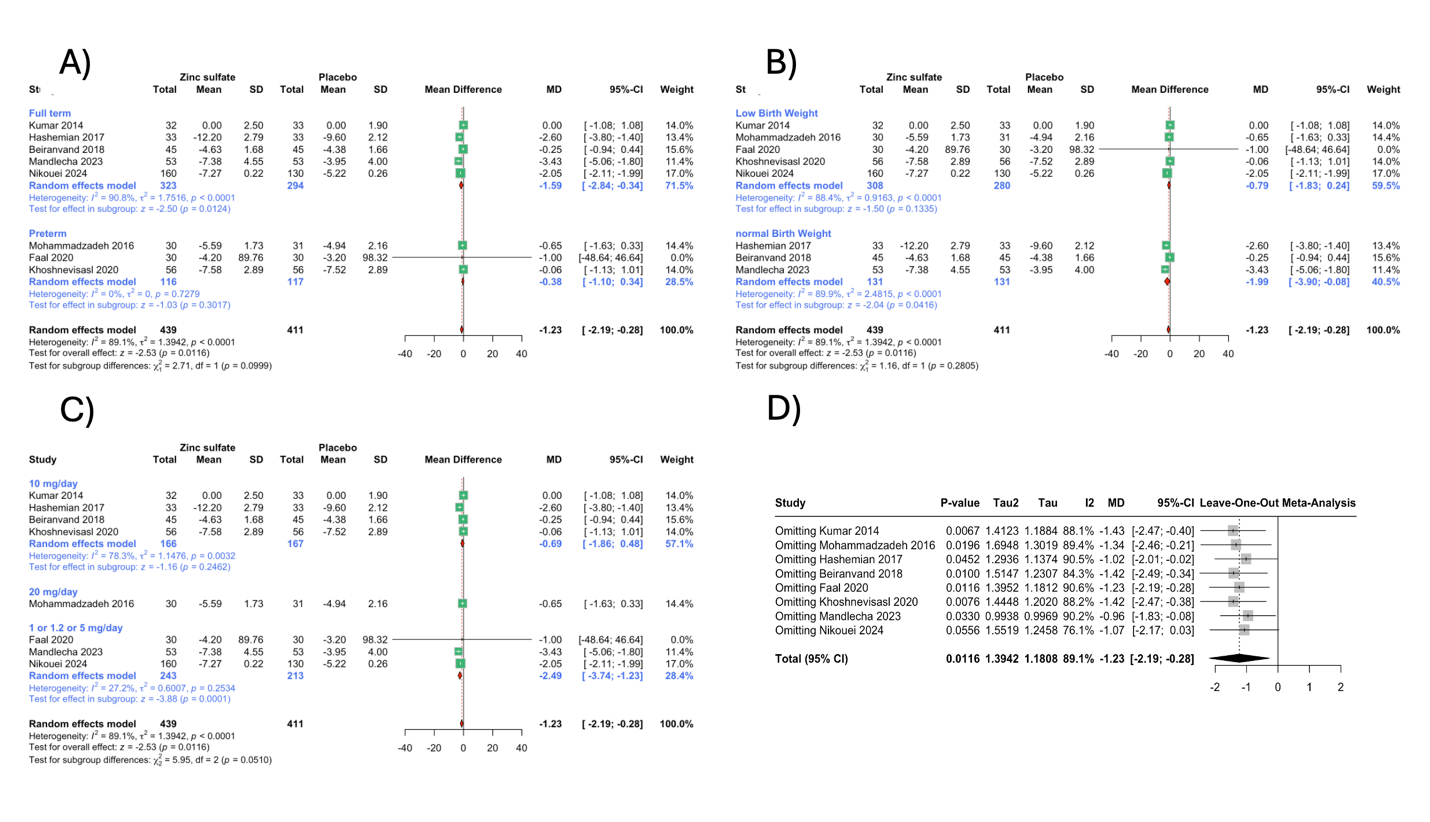
**

**Supplementary Figure 2:** Meta-analysis forest plot for change in total serum bilirubin (TSB) at 48 hours. **A**) Subgrouping according to term status. **B**) Subgrouping according to birth weight. **C**) Subgrouping according to zinc dosage. **D**) Sensitivity Leave-one-out for change of total serum bilirubin (TSB) at 48 hours (**Random effects Model). The numerical heterogeneity metrics were I² and τ² statistics.**


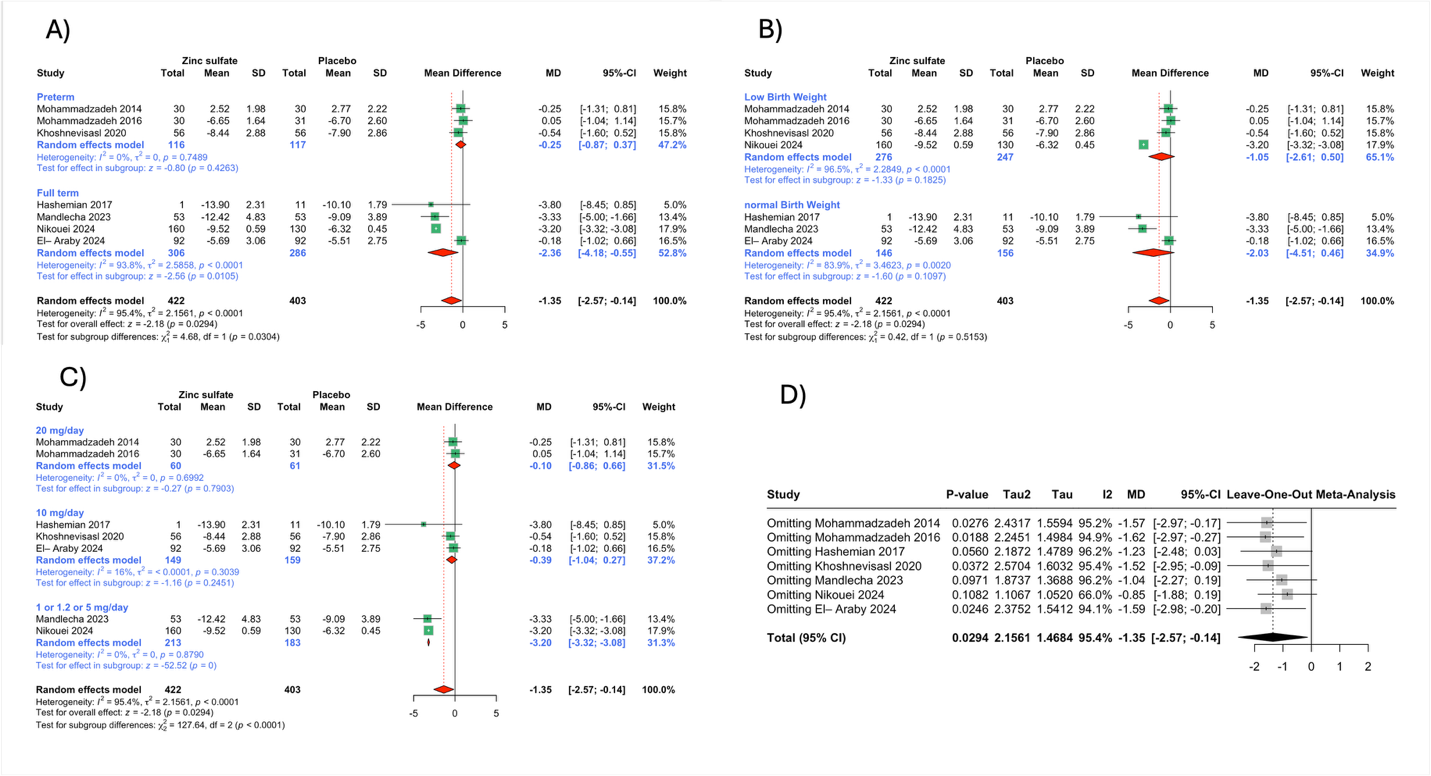


**Supplementary Figure 3:** Meta-analysis forest plot for change in total serum bilirubin (TSB) at 72 hours. **A**) Subgrouping according to term status. **B**) Subgrouping according to birth weight. **C**) Subgrouping according to zinc dosage. **D**) Sensitivity Leave-one-out for change of total serum bilirubin (TSB) at 72 hours (**Random effects Model). The numerical heterogeneity metrics were I² and τ² statistics.**


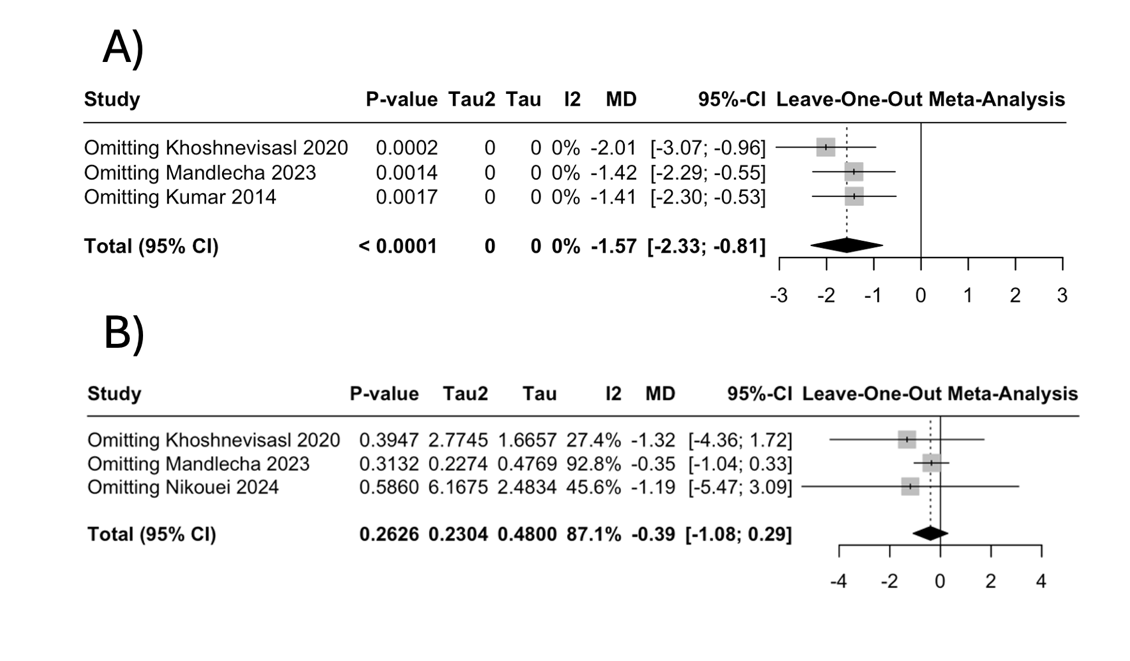


**Supplementary Figure 4:** Sensitivity Leave-one-out for **A)** mean change in total serum bilirubin after 4 days and **B)** hospital stay duration (**Random effects Model). The numerical heterogeneity metrics were I² and τ² statistics.**


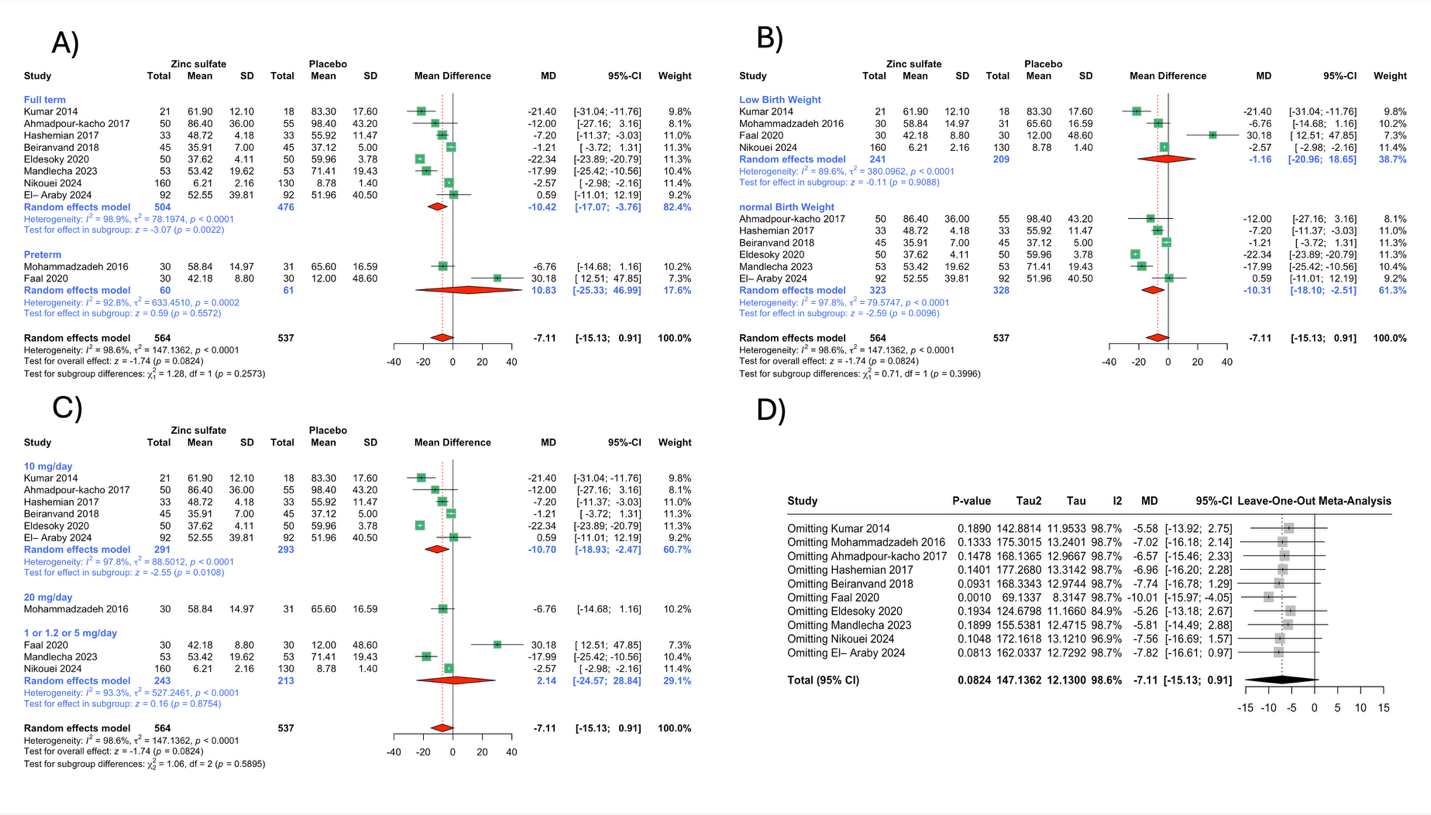


**Supplementary Figure 5:** Meta-analysis forest plot for change in phototherapy duration. **A**) Subgrouping according to term status. **B**) Subgrouping according to birth weight. **C**) Subgrouping according to zinc dosage. **D**) Sensitivity Leave-one-out for change in phototherapy duration (**Random effects Model). The numerical heterogeneity metrics were I² and τ² statistics.**


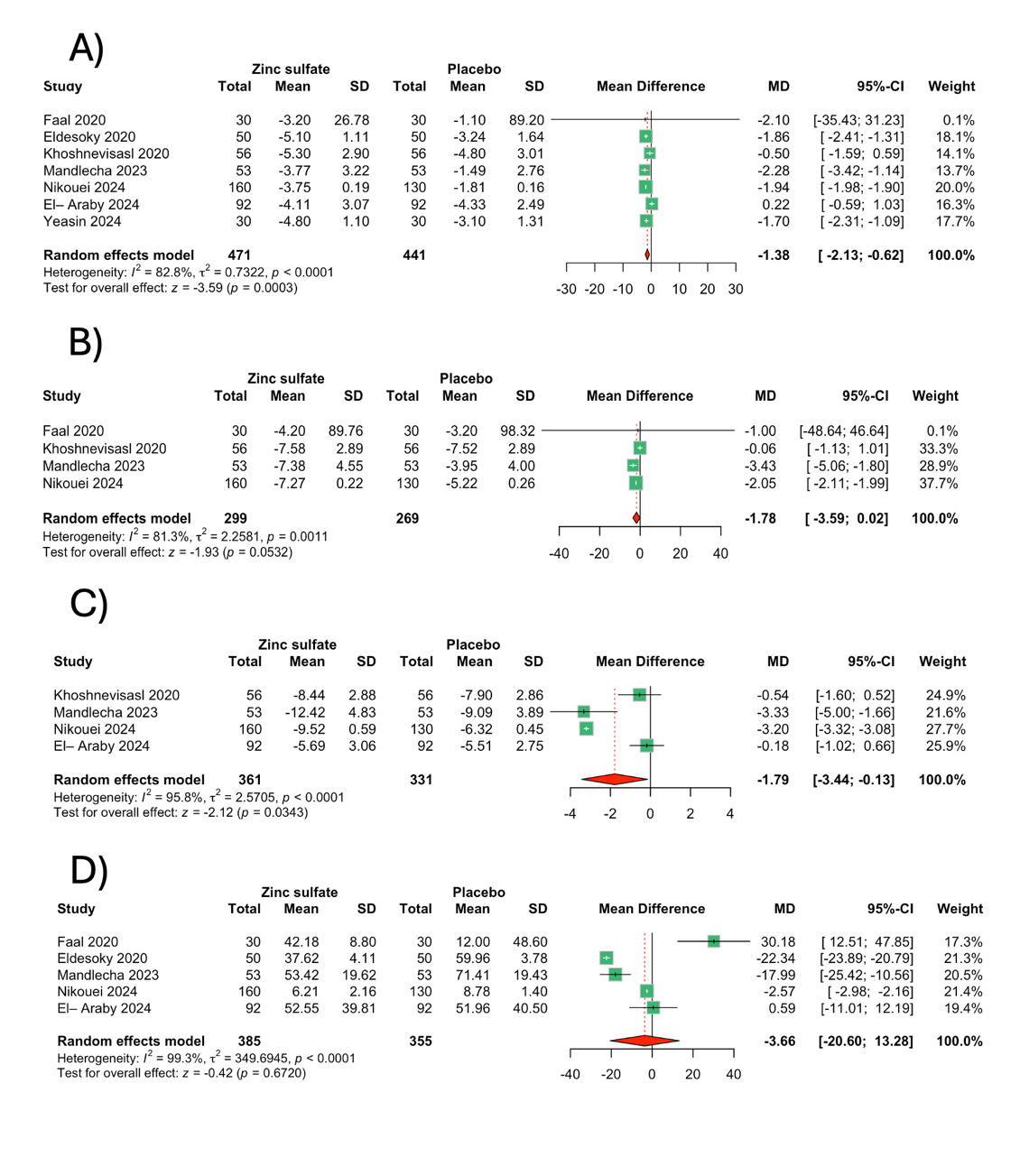


**Supplementary Figure 6:** Sensitivity analysis after excluding unclear/high-risk of bias studies. **A)** Change in total serum bilirubin (TSB) at 24 hours, **B)** Change in total serum bilirubin (TSB) at 48 hours, **C)** Change in total serum bilirubin (TSB) at 72 hours, **D)** phototherapy duration (**Random effects Model). The numerical heterogeneity metrics were I² and τ² statistics.**
